# Impure Water Makes Impure Ice

**Published:** 1882-10

**Authors:** 


### Impure Water Makes Impure Ice.

Prof. Raphael Pumpelly, of the United States Geological Survey, expresses himself as follows in the *Sanitarian*:

1. Water in freezing frees from substances which in solution with it give a fluid of greater specific gravity than water alone; but it still retains, even of these, more or less, entangled in the ice.

Substances floating in suspension are entangled in the ice, and to this the microscopic low forms of life, among which are the infectious germs, form no exceptions.

2d. Ordinary organic impurities are merely preserved from putrefaction during the period of their existence in a frozen condition. Freezing is only a temporary arresting of the processes of decomposition, and these are resumed when the requisite temperature is again attained.

With regard to the effect of freezing on the specific characters of these low forms of life (the germs of infectious disease) we know that as germs they withstand a pretty good baking and freezing; but I think it is not yet settled whether they lose their infectious character in these processes.

It certainly seems to me that no less care is necessary in maintaining the purity of ice than of ordinary drinking water; indeed, one should suppose that greater care would be necessary, for the following reason.

While, during the warmer season, the dead organic matter carried in streams and ponds is rapidly decomposed, or used up in nourishing plant and animal life, during the cold weather it tends to accumulate, and owing to its lightness, it may well happen that the ice in forming may entangle so much of it and of bacteria germs (those of infectious diseases) as to render the ice much more contaminated than a corresponding amount of drinking water from the same scource.

DISEASE IN THE SCHOOL-ROOM.—It is a sorrowful fact, and shameful to the management, that our public schools are frequently the chief agencies for the spread of epidemic and contagious diseases, and thus is planted the seeds of disease while cultivating the intellectual faculties.

